# Complete Genome of the Chitin-Degrading Bacterium, *Paenibacillus xylanilyticus* W4

**DOI:** 10.1093/gbe/evz241

**Published:** 2019-10-31

**Authors:** Weifang Liao, Pulin Liu, Weijie Liao, Lihong Miao

**Affiliations:** 1 School of Biology and Pharmaceutical Engineering, Wuhan Polytechnic University, P.R. China; 2 School of Life Sciences, Tsinghua University, Beijing, P.R. China

**Keywords:** *Paenibacillus xylanilyticus*, genome, phylogeny, chitinase

## Abstract

Chitinases possess an extraordinary ability to directly hydrolyze highly insoluble chitin polymers to low-molecular-weight chito-oligomers, which possess particular biological functions, such as elicitor action and antitumor activity. A novel strain, *Paenibacillus xylanilyticus* W4, which was isolated from soil, showed strong chitin degradation activity. Here, we first reported the complete genome information of *P*. *xylanilyticus*. *Paenibacillus xylanilyticus* W4 contains a 5,532,141 bp single circular chromosome with 47.33% GC content. The genome contains 5,996 genes, including 39 rRNA- and 109 tRNA-coding genes. Phylogenetic analysis and Genome-to-Genome Distance revealed its taxonomic characterization into a separate family. Six glycoside hydrolase 18 (GH18) and 2 GH23 enzymes involved in chitin degradation. Although many of the chitinases were conserved in *Paenibacillus*, several GH18 chitinases share high similarity with *Bacillus circulans*. The genome information provided here could benefit for understanding the chitin-degrading properties of *P*. *xylanilyticus* as well as its potential application in biotechnological and pharmaceutical fields.

## Introduction

Chitin is a major component of the cell walls of filamentous fungi and basidiomycetes, and is widely found in the outer shells of arthropod exoskeletons and crustaceans (Aam et al. 2010). The annual amount of chitin produced in nature exceeds 100 billion tons, and it is the second largest organic matter produced after cellulose (Das et al. 2010).

Chitinase can catalyze the hydrolysis of chitin, which is widely found in higher animals and plants, microorganisms, and certain viruses (Dahiya et al. 2006). Chitinase produced by microorganisms can degrade fungal cell walls and insect chitin coats and is applied in plant pest control (Kishore et al. 2005). Chitosan oligosaccharides formed by the degradation of chitin have antibacterial and anticancer properties and regulating immunity; thus, have broad application prospects in health products and pharmaceutical industry (Bhattacharya et al. 2007).

Many chitinase-producing species have been isolated from various environments, and their chitinases have been identified (Krithika and Chellaram 2016; Gasmi et al. 2019). Most known chitinases belong to GH18 and 19 families. GH18 chitinases exists widely in many organisms from microbes to human. Their catalytic domain has an absolutely conserved (β/α)_8_-barrel structure (Synstad et al. 2004). Different from GH18 family, GH19 chitinases are found only in plants and some bacteria (Ueda et al. 2003). Their steric structures reveal high α-helical contents and have an active topological structures (Kezuka et al. 2006; Huet et al. 2008). In addition, a few chitinases belonging to GH23 and GH48 families have also been discovered in recent years.

However, the chitin degradation activity of *Paenibacillus xylanilyticus* has not been investigated. Moreover, the genome information of *P*. *xylanilyticus* remains obscure. To provide a genome resource for investigations of the chitin degradation ability of *P. xylanilyticus*, and for the utilization of this bacterium, here we present the complete genome assembly, and genome annotation of *P*. *xylanilyticus* W4, a novel chitin-degrading strain isolated from soil.

## Materials and Methods

### Growth Conditions and Genomic DNA Isolation

*Paenibacillus* *xylanilyticus* W4, a novel chitin-degrading strain, was isolated from soil. *Paenibacillusxylanilyticus* W4 was grown on an M9 agar plate culture at 37 °C for 3 days (Dvorak and Lorenzo 2018). A single colony was then cultivated in 50 mL of the M9 medium overnight. Cells were collected and genomic DNA was extracted using a Qiagen QIAamp DNA Mini Kit (Qiagen, Germany). The quantity and quality of the extraction were checked by agarose gel electrophoresis and the Nanodrop method and followed by Qubit quantification.

### Genome Sequencing, Assembly, and Annotation

A PacBio 10-kb sequencing library was constructed with a SMRTbell template prep kit 1.0. The final library was processed for sequencing in a single-molecule real-time (SMRT) cell using P6 polymerase and C4 chemistry on a PacBio instrument. The PacBio hierarchical genome assembly process (HGAP) version 2.0 was used for the de novo assembly of the sequence reads. Given that the BluePippin Size Selection (>20,000) protocol filters out small DNA fragments, such as plasmids, Solexa paired-end sequencing data were also obtained by using the Illumina HiSeq2000. To trace the presence of any plasmid, we mapped the filtered Illumina reads by using CLCbio wb8.0 (www.clcbio.com) to the bacterial plasmid database (http://www.ebi.ac.uk/genomes/plasmid.html).

Functional annotation was checked by rapid annotation using Subsystem Technology (RAST, version 3.0, Overbeek et al. 2008), Cluster of Orthologous Group (COG, Tatusov et al. 2000), Gene Ontology (GO, Aam et al. 2010), and Kyoto Encyclopedia of Genes and Genomes (KEGG, Moriya et al. 2007). rRNAs and tRNAs were predicted by using Barrnap 0.4.2 and tRNAscan-SE (version 1.3.1, Lowe and Eddy 1997), respectively. CRISPR elements were identified using CRISPR finder (Grissa et al. 2007).

### Phylogenetic Analysis and Genome-to-Genome Distance

The 16S rRNA genes of various *Paenibacillus* strains and neighboring families were downloaded from the National Centre for Biotechnology Information (NCBI), and were aligned by using the ClustalW module of BIOEDIT sequence alignment tool (version 7.1.3.0). A phylogenic tree based on the resulting alignment was then constructed by the Neighbor-Joining and maximum likelihood methods with the MEGA X version 10.1 program package (Kumar et al. 2018). In silico DNA–DNA hybridization (DDH) values among *Paenibacillus* members and other Paenibacillaceae species members were calculated by using the Genome-To-Genome Distance Calculator (GGDC) server (Auch et al. 2010).

### *P. xylanilyticus* W4 Chitinases

Putative CAZymes in *P. xylanilyticus* W4 proteome were identified using the CAZy annotation pipeline (Lombard et al. 2014). The functional domains involved in chitin degradation were retrieved. The proteins of *P. xylanilyticus* W4 were then compared with the sequences from the CAZy protein database by using the BLASTp tool (*E*-value cutoff of 1e^−5^). Then, chitinases from *P. xylanilyticus* W4 were subjected to the BLASTp against chitinase sequences from the CAZy database, and the sequences with >70% similarity were retrieved. The chitinases from *P. xylanilyticus* W4 were cut into the domains and were aligned by using MUSCLE incorporated in MEGA X version 10.1 (Kumar et al. 2018). Furthermore, an evolutionary tree was generated by the maximum likelihood method (model: Jones-Taylor-Thornton; bootstrap: 100).

## Results and Discussion

### *P. xylanilyticus* W4 Genome Features

The genome sequence of *P. xylanilyticus* W4 was obtained using a combination of PacBio and Illumina HiSeq2000 technologies. The whole-genome sequencing by PacBio system yielded a total of 62,631 reads with a mean read length of 8.46 kb and 95.78-fold genome coverage. DNA sequencing by Illumina HiSeq2000, generated a total of 9,653,276 reads, with 126.32 bp average read length, and 220.42-fold genome coverage. After assembly, *P. xylanilyticus* W4 showed single circular chromosome (supplementary fig. S1, Supplementary Material online). The overall *P. xylanilyticus* W4 genome feature is shown in table 1. The coding proteins identified were classified into 19 functional categories according to the clusters of the orthologous groups (COG) of proteins (supplementary fig. S1, Supplementary Material online). Among all the categories, the carbohydrate transport and metabolism category (G, 15.29%) was the largest.

**Table 1:** Genome Features of *Paenibacillus xylanilyticus* W4

Features	Chromosome
Genome size (bp)	5,532,141
GC content (%)	47.33
Gene number	5,996
Gene average length (bp)	922
rRNA genes	16
tRNA genes	109
CRISPR	0
Phage	3

### Phylogenetic Position of *P. xylanilyticus* W4

The phylogenic tree based on 16S rRNA sequences of *Paenibacillus* and related Paenibacillaceae members showed that *P. xylanilyticus* 16S rRNA forms a separate branch distinct from other families (supplementary fig. S2, Supplementary Material online). The in silico DDH values for the available genomes of Paenibacillaceae strains were calculated by using the GGDC server. The maximum DDH value was 63.3 with *Paenibacillusillinoisensis* E3, suggesting that *P. xylanilyticus* is not the same species as other Paenibacillaceae members and has the closest relationship with *P. illinoisensis* (supplementary table S1, Supplementary Material online).

### *P. xylanilyticus* W4 Chitinases

A total of 362 proteins of *P. xylanilyticus* W4 were annotated with various combinations of CAZy domains (supplementary fig. S3, Supplementary Material online). Six glycoside hydrolase 18 (GH18) and 2 GH23 enzymes involved in chitin degradation.

**Fig. 1.**
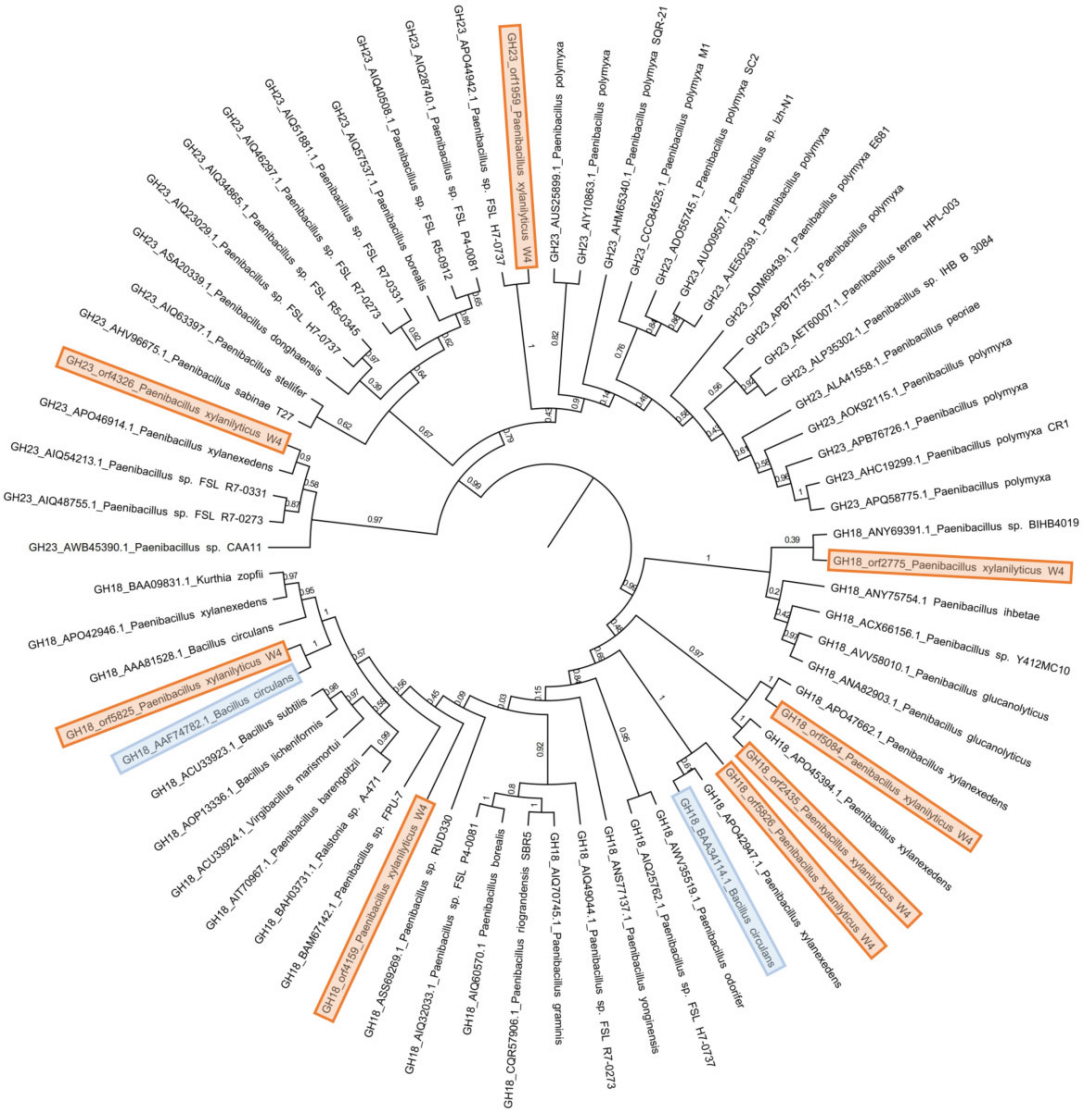
—Phylogeny of *Paenibacillus xylanilyticus* GH18 and GH23 chitinases. *P. xylanilyticus* chitinases are colored in orange. *P. xylanilyticus* homolog is colored in blue. Bootstrap values of the tree nodes are marked.

To understand the provenance of these chitinases, we subjected their sequences to BLASTp against the Nr database. The CAZy-Nr database for GH18 and GH23 domains were retrieved and subjected to BLASTClust at 70% identity. All of these GH18 and GH23 domains along with *P. xylanilyticus* chitinase domains were aligned using MUSCLE incorporated in MEGA X version 10.1 and maximum likelihood phylogeny with 100 bootstrap values was constructed. The tree obtained was divided into two major clades, where the clade at the root was composed of all GH18 sequences and the other was composed of all GH23 domains (fig. 1).

The representative chitinases from different branches show close relations with chitinases of different species of bacteria suggesting the gain and loss of the domains. In the GH23 clade, the GH23 domains orf1959 and orf4326 of *P. xylanilyticus* share the clade with other *Paenibacillus* species. The closest identified homologs of the GH18 clade have representation from diverse taxa such as *Paenibacillus*, *Bacilli*, *Ralstonia*, *Kurthia*, and *Virgibacillus* members. Among the GH18 domains of *P. xylanilyticus* W4, orf2775, orf4159, orf5084, and orf5826 share sister branches with other *Paenibacillus* species such as *P. xylanexedens*, *Paenibacillus* sp. BIHB4019, and *Paenibacillus* sp. FPU-7. Interestingly, orf2435 and orf5825 group with *Bacilluscirculans* suggestive of possible horizontal transfer.

## Supplementary Material

Supplementary data are available at *Genome Biology and Evolution* online.

## Supplementary Material

evz241_Supplementary_Data

## Notes

**Data deposition:** This project has been deposited at GenBank under the accessions MN597078-MN597085.

## References

[evz241-B1] Aam BB, et al 2010 Production of chitooligosaccharides and their potential applications in medicine. Mar Drugs. 8(5):1482–1517. 20559485 10.3390/md8051482 PMC2885077

[evz241-B3] Auch AF, Jan M, Klenk HP, Goker M. 2010 Digital DNA–DNA hybridization for microbial species delineation by means of genome-to-genome sequence comparison. Stand Genomic Sci. 2(1):117–134. 21304684 10.4056/sigs.531120 PMC3035253

[evz241-B4] Bhattacharya D, Nagpure A, Gupta RK. 2007 Bacterial chitinases: properties and potential. Crit Rev Biotechnol. 27(1):21–28. 17364687 10.1080/07388550601168223

[evz241-B5] Dahiya N, Tewari R, Hoondal GS. 2006 Biotechnological aspects of chitinolytic enzymes: a review. Appl Microbiol Biotechnol. 71(6):773–782. 16249876 10.1007/s00253-005-0183-7

[evz241-B6] Das SN, Sarma P, Neeraja C, Malati N, Podile AR. 2010 Members of *Gammaproteobacteria* and *Bacilli* represent the culturable diversity of chitinolytic bacteria in chitin-enriched soils. World J Microbiol Biotechnol. 26(10):1875–1881.

[evz241-B7] Dvorak P, Lorenzo V. 2018 Refactoring the upper sugar metabolism of *Pseudomonas putida* for co-utilization of cellobiose, xylose, and glucose. Metab Eng. 48:94–108. 29864584 10.1016/j.ymben.2018.05.019

[evz241-B8] Kezuka Y, et al 2006 Structural studies of a two-domain chitinase from *Streptomyces griseus* HUT6037. J Mol Biol. 358(2):472–484. 16516924 10.1016/j.jmb.2006.02.013

[evz241-B9] Kishore GK, Pande S, Podile AR. 2005 Phylloplane bacteria increase seedling emergence, growth and yield of field grown groundnut (*Arachis hypogaea* L.). Lett Appl Microbiol. 40(4):260–268. 15752215 10.1111/j.1472-765X.2005.01664.x

[evz241-B10] Krithika S, Chellaram C. 2016 Isolation, screening, and characterization of chitinase producing bacteria from marine wastes. Int J Pharm Pharmac Sci. 8(5):34–36.

[evz241-B11] Kumar S, Stecher G, Li M, Knyaz C, Tamura K. 2018 MEGA X: molecular evolutionary genetics analysis across computing platforms. Mol Biol Evol. 35(6):1547–1549. 29722887 10.1093/molbev/msy096 PMC5967553

[evz241-B12] Lombard V, Golaconda Ramulu H, Drula E, Coutinho PM, Henrissat B. 2014 The carbohydrate-active enzymes database (CAZy) in 2013. Nucleic Acids Res. 42(D1):D490–D495. 24270786 10.1093/nar/gkt1178 PMC3965031

[evz241-B13] Lowe TM, Eddy SR. 1997 tRNAscan-SE: a program for improved detection of transfer RNA genes in genomic sequence. Nucleic Acids Res. 25(5):955–964. 9023104 10.1093/nar/25.5.955 PMC146525

[evz241-B14] Moriya Y, Itoh M, Okuda S, Yoshizawa AC, Kanehisa M. 2007 KAAS: an automatic genome annotation and pathway reconstruction server. Nucleic Acids Res. 35(Web Server):W182–185. 17526522 10.1093/nar/gkm321 PMC1933193

[evz241-B15] Gasmi M, et al 2019 Chitinolytic actinobacteria isolated from an Algerian semi-arid soil: development of an antifungal chitinase-dependent assay and GH18 chitinase gene identification. Ann Microbiol. 69(4):395–405.

[evz241-B16] Grissa I, Vergnaud G, Pourcel C. 2007 CRISPRFinder: a web tool to identify clustered regularly interspaced short palindromic repeats. Nucleic Acids Res. 35(Web Server):W52–W57. 17537822 10.1093/nar/gkm360 PMC1933234

[evz241-B17] Huet J, et al 2008 X-ray structure of papaya chitinase reveals the substrate binding mode of glycosyl hydrolase family 19 chitinases. Biochemistry 47(32):8283–8291. 18636748 10.1021/bi800655u

[evz241-B18] Overbeek RA, et al 2008 The RAST server: rapid annotations using subsystems technology. BMC Genomics 9:75. 18261238 10.1186/1471-2164-9-75 PMC2265698

[evz241-B19] Synstad B, et al 2004 Mutational and computational analysis of the role of conserved residues in the active site of a family 18 chitinase. Eur J Biochem. 271(2):253–262. 14717693 10.1046/j.1432-1033.2003.03923.x

[evz241-B20] Tatusov RL, Galperin MY, Natale DA, Koonin EV. 2000 The COG database: a tool for genome-scale analysis of protein functions and evolution. Nucleic Acids Res. 28(1):33–36. 10592175 10.1093/nar/28.1.33 PMC102395

[evz241-B21] Ueda M, et al 2003 A novel type of family 19 chitinase from Aeromonassp. No. 10S-24. Cloning, sequence, expression, and the enzymatic properties. Eur J Biochem. 270(11):2513–2520. 12755707 10.1046/j.1432-1033.2003.03624.x

