# Supplementary material for: Complete Genome of the Chitin-Degrading Bacterium, Paenibacillus xylanilyticus W4
Source: Genome Biol Evol. 2019 Oct 31;11(11):3252–5. doi: 10.1093/gbe/evz241 (PMC6865854; doi:10.1093/gbe/evz241)
Supplement: evz241_Supplementary_Data [file evz241_supplementary_data.doc]

**Table S1**

The in silico DDH values for genomes of Paenibacillaceae strains.

| Query genome | Reference genome | accession number | DDH | Distance | Prob. DDH >= 70% |
| --- | --- | --- | --- | --- | --- |
| *P. xylanilyticus* W4 | *P. larvae* | NZ_CP019687.1 | 33.7 | 0.1238 | 0.43 |
| *P. xylanilyticus* W4 | *P. barengoltzii* | NZ_ASSZ00000000.1 | 25.9 | 0.1678 | 0.01 |
| *P. xylanilyticus* W4 | *P. alvei* DSM 29 | NZ_AMBZ00000000.1 | 35.3 | 0.1168 | 0.73 |
| *P. xylanilyticus* W4 | *P. mucilaginosus* 3016 | NC_016935.1 | 36.3 | 0.1127 | 0.99 |
| *P. xylanilyticus* W4 | *P. massiliensis* 2301065 | NZ_ARIL00000000.1 | 20.5 | 0.2145 | 0 |
| *P. xylanilyticus* W4 | *P. validus* | NZ_BIMH00000000.1 | 32.1 | 0.1312 | 0.24 |
| *P. xylanilyticus* W4 | *P. humicus* | NZ_BIMD00000000.1 | 31.8 | 0.1326 | 0.22 |
| *P. xylanilyticus* W4 | *P. Illinoisensis* E3 | GCA_003224455.1 | 63.3 | 0.0462 | 62.56 |
| *P. xylanilyticus* W4 | *P. thiaminolyticus* | GCA_002161855.1 | 32.7 | 0.1286 | 0.3 |
| *P. xylanilyticus* W4 | *P. lautus* | NZ_CP032412.1 | 26.2 | 0.1653 | 0.02 |
| *P. xylanilyticus* W4 | *P. pabuli NBRC 13638* | NZ_BCNM00000000.1 | 23.6 | 0.1853 | 0 |
| *P. xylanilyticus* W4 | *P. macerans* | NZ_JMQA00000000.1 | 26.9 | 0.1605 | 0.03 |
| *P. xylanilyticus* W4 | *P. sabinae* T27 | NZ_CP004078.1 | 26.2 | 0.1655 | 0.02 |
| *P. xylanilyticus* W4 | *P. glucanolyticus* 5162 | NZ_CP015286.1 | 24.5 | 0.1779 | 0.01 |
| *P. xylanilyticus* W4 | *P. senegalensis* JC66 | NZ_CAES00000000.1 | 34.5 | 0.1203 | 0.56 |
| *P. xylanilyticus* W4 | *P. pini* JCM 16418 | NZ_BAVZ00000000.1 | 23.3 | 0.188 | 0 |
| *P. xylanilyticus* W4 | *P. lentimorbus* | NZ_ANAT00000000.1 | 31.8 | 0.1328 | 0.21 |
| *P. xylanilyticus* W4 | *P. forsythiae* T98 | NZ_ASSC00000000.1 | 23.1 | 0.1894 | 0 |
| *P. xylanilyticus* W4 | *P. zanthoxyli* JH29 | NZ_ASSD00000000.1 | 26.6 | 0.1625 | 0.02 |
| *P. xylanilyticus* W4 | *P. graminis* | NZ_CP009287.1 | 25.2 | 0.1729 | 0.01 |
| *P. xylanilyticus* W4 | *P. durus* DSM1735 | NZ_CP009288.1 | 29.4 | 0.1454 | 0.08 |
| *P. xylanilyticus* W4 | *P. panacisoli* DSM 21345 | NZ_AUFO00000000.1 | 20.3 | 0.2167 | 0 |
| *P. xylanilyticus* W4 | *P. pinihumi* DSM 23905 | NZ_AULX00000000.1 | 21.7 | 0.2024 | 0 |
| *P. xylanilyticus* W4 | *P. pasadenensis* DSM 19293 | NZ_AULW00000000.1 | 35.6 | 0.1158 | 0.79 |
| *P. xylanilyticus* W4 | *P. harenae* DSM 16969 | NZ_AULV00000000.1 | 21.2 | 0.2075 | 0 |
| *P. xylanilyticus* W4 | *P. assamensis* DSM 18201 | NZ_AULU00000000.1 | 23.1 | 0.1891 | 0 |
| *P. xylanilyticus* W4 | *P. taiwanensis* DSM 18679 | NZ_AULE00000000.1 | 22.1 | 0.1987 | 0 |
| *P. xylanilyticus* W4 | *P. fonticola* DSM 21315 | NZ_ARMT00000000.1 | 20.5 | 0.2148 | 0 |
| *P. xylanilyticus* W4 | *P. ginsengihumi* DSM 21568 | NZ_ARKW00000000.1 | 29.6 | 0.1441 | 0.09 |
| *P. xylanilyticus* W4 | *P. daejeonensis* DSM 15491 | NZ_ARKE00000000.1 | 21.6 | 0.203 | 0 |
| *P. xylanilyticus* W4 | *P. popilliae* ATCC 14706 | NZ_BALG00000000.1 | 29.6 | 0.1444 | 0.09 |
| *P. xylanilyticus* W4 | *P. terrigena* DSM 21567 | NZ_ARGP00000000.1 | 21.7 | 0.2018 | 0 |
| *P. xylanilyticus* W4 | *P.koleovorans* | NZ_BIMA00000000.1 | 31.2 | 0.1356 | 0.17 |
| *P. xylanilyticus* W4 | *P. kobensis* | NZ_BILZ00000000.1 | 26.6 | 0.1627 | 0.02 |
| *P. xylanilyticus* W4 | *P. glycanilyticus* | NZ_BILY00000000.1 | 24.2 | 0.1807 | 0.01 |
| *P. xylanilyticus* W4 | *P. chitinolyticus* NBRC 15660 | NZ_BBJT00000000.1 | 30.2 | 0.1412 | 0.11 |
| *P. xylanilyticus* W4 | *P. alginolyticus* DSM 15375 | NZ_AUGY00000000.1 | 21.1 | 0.208 | 0 |
| *P. xylanilyticus* W4 | *P. sanguinis* 2301083 | NZ_ARGO00000000.1 | 20.9 | 0.2106 | 0 |
| *P. xylanilyticus* W4 | *P. sonchi* X19-5 | NZ_AJTY00000000.1 | 22.5 | 0.1946 | 0 |
| *P. xylanilyticus* W4 | *P. terrae* HPL-003 | NC_016641.1 | 26.7 | 0.1621 | 0.02 |
| *P. xylanilyticus* W4 | *P. dendritiformis* C454 | NZ_AHKH00000000.1 | 25.8 | 0.1681 | 0.01 |
| *P. xylanilyticus* W4 | *P. peoriae* | NZ_CP011512.1 | 25.3 | 0.1716 | 0.01 |
| *P. xylanilyticus* W4 | *P. hemerocallicola* KCTC 33185 | NZ_VDCQ00000000.1 | 32.4 | 0.1299 | 0.27 |
| *P. xylanilyticus* W4 | *P. xylaniclasticus* | NZ_BIML00000000.1 | 29.3 | 0.1458 | 0.08 |
| *P. xylanilyticus* W4 | *Brevibacillus borstelensis* AK1 | NZ_APBN00000000.1 | 26.7 | 0.1624 | 0.02 |
| *P. xylanilyticus* W4 | *Brevibacillus brevis NBRC* 100599 | NC_012491.1 | 28.1 | 0.1528 | 0.05 |
| *P. xylanilyticus* W4 | *Brevibacillus thermoruber* 423 | NZ_ATNE00000000.1 | 30.7 | 0.1384 | 0.14 |
| *P. xylanilyticus* W4 | *Brevibacillus panacihumi* W25 | NZ_AYJU00000000.1 | 29.2 | 0.1465 | 0.08 |

**Figure legends**

**Fig.S1** Circular genome map of *P. xylanilyticus* W4. GC skew (innermost circle), GC content (second circle), CDs on forward and reverse chains (third and fourth circles), Genomic scale (fifth circle) are shown. Different colors indicate different COG classifications. The circular representation was created by the Circos software.

**Fig.S2** Phylogenetic tree of *P. xylanilyticus* W4. The tree was constructed using MEGA X 10.1 by neighbor-joining method with Maximum Composite Likelihood method based on 16s rRNA gene sequences with 100 replications in bootstrap test.

**Fig.S3** *P. xylanilyticus* W4 CAZyme distribution. x axis shows the CAZy categories and y axis represents the quantity of proteins.


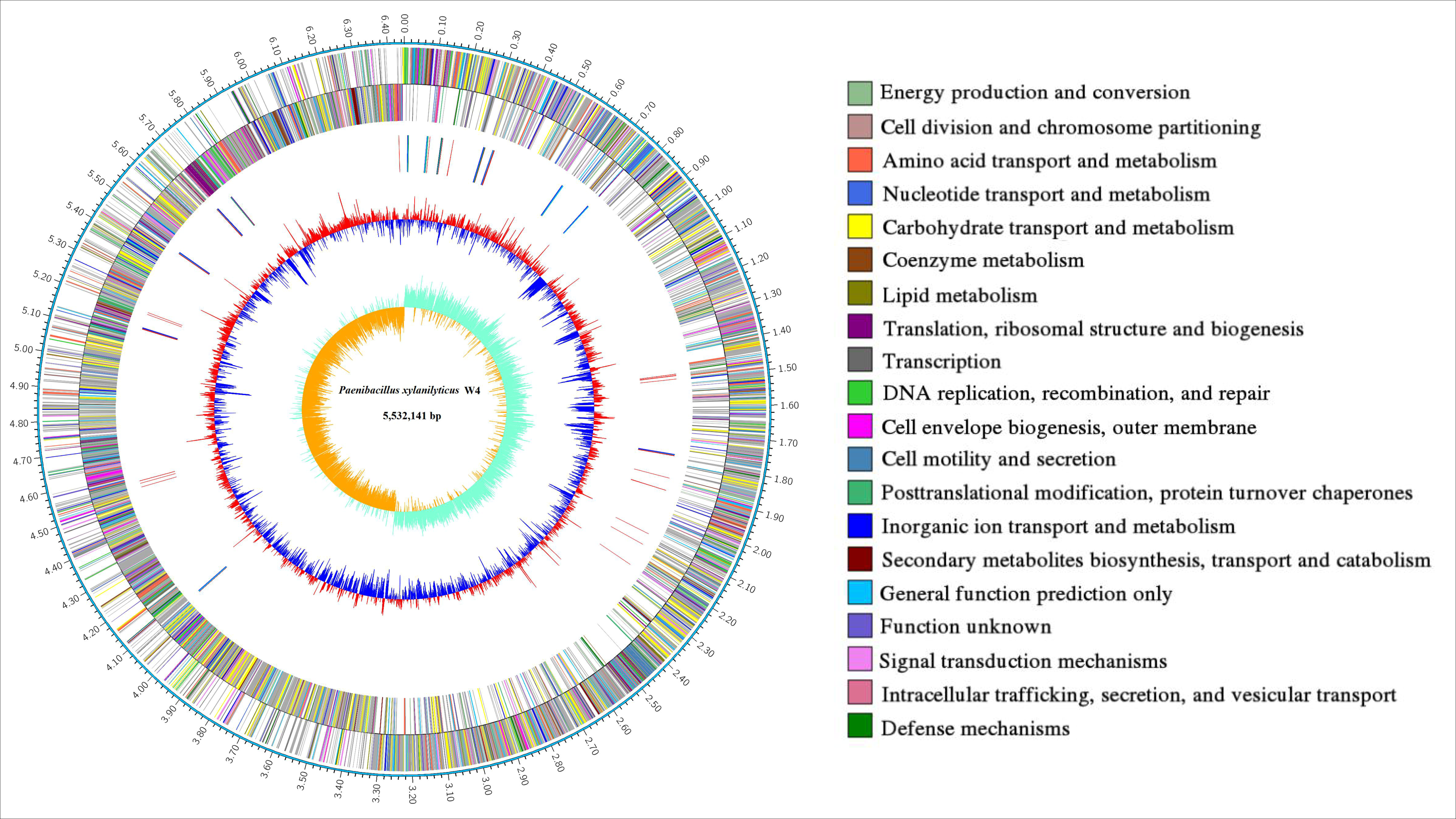


Fig.S1


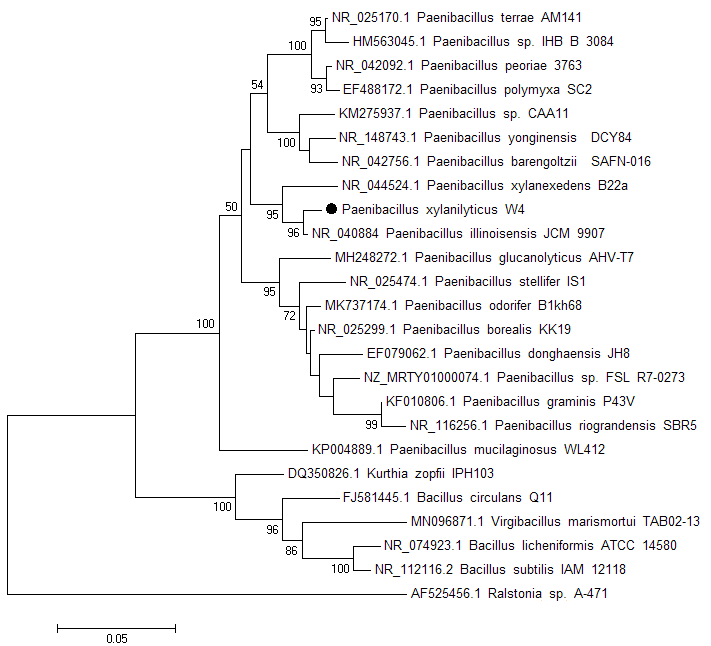


Fig.S2


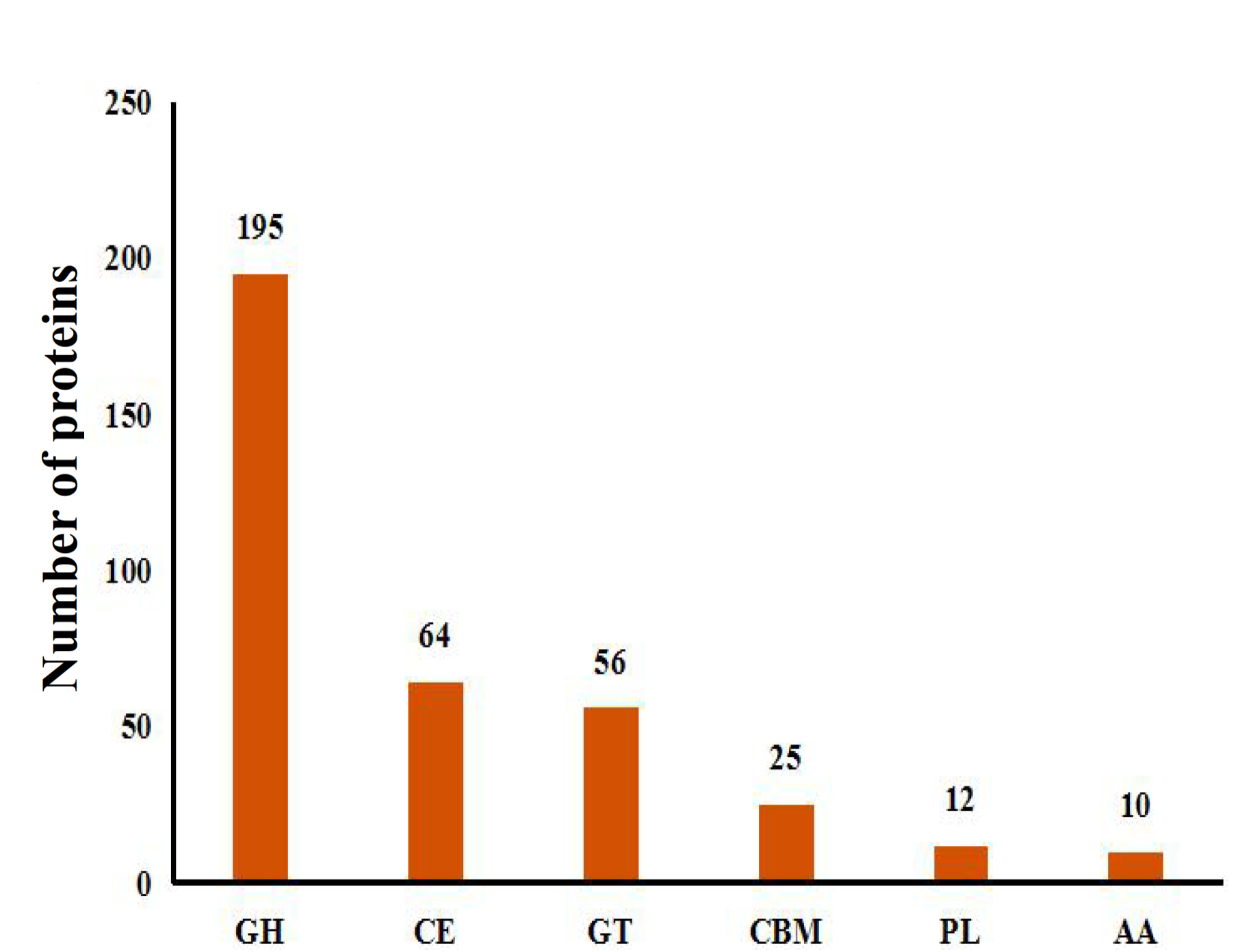


Fig.S3
